# ATP8B1 Deficiency Causes Phosphodiesterase 4‐Mediated Glucagon Resistance and Impaired Gluconeogenesis in Mouse and Human Liver

**DOI:** 10.1111/liv.70306

**Published:** 2025-08-25

**Authors:** Jung‐Chin Chang, Wietse In het Panhuis, Shu‐Hao Hsu, Suzanne Duijst, Kam S. Ho‐Mok, Jolie F. van der Heiden, Zhixiong Ying, Joris I. Erdmann, Georg F. Vogel, Norman Junge, Björn Hartleben, Pim J. Koelink, Sebastian G. van Dijk, Weng Chuan Peng, Arthur J. Verhoeven, Ronald P. J. Oude Elferink, Huey‐Ling Chen, Stan F. J. van de Graaf, Coen C. Paulusma

**Affiliations:** ^1^ Department of Biomolecular Health Sciences Faculty of Veterinary Medicine, Utrecht University Utrecht the Netherlands; ^2^ Tytgat Institute for Liver and Intestinal Research Amsterdam UMC, University of Amsterdam Amsterdam the Netherlands; ^3^ Amsterdam Gastroenterology Endocrinology Metabolism (AGEM) Research Institute Amsterdam UMC, University of Amsterdam Amsterdam the Netherlands; ^4^ Graduate Institute of Anatomy and Cell Biology National Taiwan University College of Medicine Taipei Taiwan; ^5^ Department of Surgery, Cancer Center Amsterdam Amsterdam UMC, University of Amsterdam Amsterdam the Netherlands; ^6^ Department of Pediatrics I Medical University of Innsbruck Innsbruck Austria; ^7^ Institute of Cell Biology, Biocenter Medical University of Innsbruck Innsbruck Austria; ^8^ Division for Pediatric Gastroenterology and Hepatology, Department of Pediatric Kidney, Liver, and Metabolic Diseases Hannover Medical School Hannover Germany; ^9^ Institute of Pathology Hannover Medical School Hannover Germany; ^10^ Princess Máxima Center for Pediatric Oncology Utrecht the Netherlands; ^11^ Department of Pediatrics National Taiwan University Children's Hospital Taipei Taiwan; ^12^ Department and Graduate Institute of Medical Education and Bioethics National Taiwan University College of Medicine Taipei Taiwan

**Keywords:** cholestasis, cyclic adenosine monophosphate, gluconeogenesis, glucose

## Abstract

**Background and Aims:**

Deficiency of the phospholipid transporter ATP8B1 causes infantile‐onset progressive familial intrahepatic cholestasis type I (PFIC1). Pre‐transplant PFIC1 patients often present with mild dyslipidaemia. This raises the possibility that PFIC1 patients, besides cholestasis, may also experience defects in glucose and lipid metabolism. In this study, we aimed to investigate the role of ATP8B1 in hepatic glucose and lipid metabolism using the *Atp8b1* ^*G308V/G308V*^ mutant mouse, a pre‐clinical model of PFIC1.

**Methods:**

*Atp8b1* ^G308V/G308V^ and wild‐type mice on normal chow were examined. Hepatic glucose metabolism was evaluated by oral glucose tolerance testing, quantification of fasting plasma glucose, insulin and lipids. Mechanistic studies were conducted in primary mouse hepatocytes (PMHs) and HepG2 cells overexpressing glucagon receptor (HepG2‐GCGR). The findings in the mouse model were validated in pre‐transplant livers from PFIC1 patients.

**Results:**

*Atp8b1* ^G308V/G308V^ mice showed decreased levels of fasting blood glucose, triglycerides and insulin, indicating normal insulin sensitivity and impaired hepatic glucagon response. PMHs from *Atp8b1* ^G308V/G308V^ mice exhibited reduced glucagon‐dependent cAMP levels and signalling and had increased expression of *Pde4* isoforms. Rolipram‐mediated PDE4 inhibition restored glucagon signalling. ATP8B1 knockdown HepG2‐GCGR cells also showed increased PDE4D expression and impaired glucagon signalling. Liver tissue from PFIC1 patients exhibited elevated PDE4D and reduced p‐CREB levels and very low glycogen content.

**Conclusions:**

ATP8B1 deficiency causes upregulation of PDE4D in the liver of *Atp8b1* ^G308V/G308V^ mice and PFIC1 patients. PDE4D‐mediated glucagon resistance impairs gluconeogenesis and stimulates compensatory glycogenolysis in *Atp8b1* ^G308V/G308V^ mice and PFIC1 patients. Our study reveals novel metabolic insights of ATP8B1 deficiency in PFIC1 patients.

**Summary**

- ATP8B1 deficiency causes progressive familial intrahepatic cholestasis type 1 (PFIC1) and is characterised by infantile‐onset cholestasis but also dyslipidaemia.
- ATP8B1 deficiency in mice leads to upregulation of the cAMP‐degrading phosphodiesterase 4D to cause glucagon resistance and impaired gluconeogenesis.
- Livers of PFIC1 patients showed increased PDE4D and reduced p‐CREB immunoreactivity and extremely low glycogen content, indicating aberrant glucose metabolism.

**Abbreviations**
cAMP: cyclic adenosine monophosphate
G6PC1: glucose‐6‐phosphatase catalytic subunit 1
GCGR: glucagon receptor
PDE: phosphodiesterase
PFIC1: progressive familial intrahepatic cholestasis type 1
PMH: primary mouse hepatocyte
WT: wild type

## 1 Introduction

Progressive familial intrahepatic cholestasis type 1 (PFIC1) is an autosomal recessive cholestatic liver disease caused by mutations in *ATP8B1*, which encodes a phospholipid translocase with a broad substrate specificity [1, 2]. Patients typically present during the first few years of life with cholestasis, accompanied by severe pruritus and progressive liver damage that can lead to cirrhosis [3, 4]. In addition to cholestasis, PFIC1 patients often develop extrahepatic symptoms and signs, such as hearing impairment, growth failure, abnormal sweat production, pulmonary infection and gastrointestinal problems [3, 5, 6]. Liver transplantation can alleviate cholestasis, but extrahepatic manifestations may persist or even worsen. Notably, 60%–90% of post‐transplant PFIC1 patients develop hepatic steatosis or steatohepatitis [7, 8], a condition recently linked to intestinal deficiency of ATP8B1 [9]. This suggests that pre‐transplant PFIC1 patients might have impaired glucose and lipid metabolism, but the metabolic phenotypes could be masked or complicated by concurrent cholestasis. Consistent with this idea, pre‐transplant PFIC1 patients frequently present with hypertriglyceridaemia and low high‐density lipoprotein levels, but the underlying cause remains unclear [10, 11].

Glucagon regulates the blood glucose level and fatty acid oxidation [12]. During fasting, low glucose levels trigger glucagon release from pancreatic α‐cells into the hepatic portal system. Glucagon binds to the glucagon receptor (GCGR), activating Gαs and stimulating adenylyl cyclases to produce cAMP. cAMP activates protein kinase A (PKA), which phosphorylates the cAMP response element‐binding protein (CREB) to induce gluconeogenic genes, including glucose‐6‐phosphatase (*G6PC1*), and via a phosphorylation cascade promoting glycogenolysis. Additionally, GCGR‐cAMP signalling activates phosphorylation of inositol 1,4,5‐trisphosphate receptors (InsP3Rs), triggering Ca^2+^ release that enhances mitochondrial fatty acid oxidation to support gluconeogenesis [13, 14].

The steady‐state concentration of cAMP is regulated via its production by adenylyl cyclases and its degradation by phosphodiesterases (PDEs). PDEs also play a crucial role in maintaining the compartmentalisation of cAMP signalling microdomains, ensuring the independence and specificity of GPCR signalling [15]. Mammalian PDEs comprise 11 subfamilies with differing substrate specificities and tissue distributions [16]. In the liver, members of the PDE3 and PDE4 subfamilies are key regulators of gluconeogenesis and glycogenolysis [17]. Notably, elevated PDE4 expression has been associated with various liver pathologies, including cholestasis, alcoholic liver disease (ALD) and metabolic dysfunction‐associated steatotic liver disease [18, 19, 20].

In the present study, we investigate the role of ATP8B1 in hepatic glucose metabolism using the pre‐clinical PFIC1 mouse model, the *Atp8b1* ^*G308V/G308V*^ mutant mouse [21]. Unlike PFIC1 patients, these mice do not develop cholestasis unless challenged with cholate, allowing us to examine ATP8B1 deficiency without the confounding effects of liver injury. We found that ATP8B1 deficiency impaired hepatic glucose production during fasting, due to increased expression of cAMP‐degrading PDE4D, which suppresses glucagon‐mediated cAMP signalling and impairs gluconeogenesis. Importantly, immunohistochemistry of native livers of PFIC1 patients showed elevated PDE4D, reduced p‐CREB levels and near‐complete absence of hepatic glycogen, strongly suggesting that increased PDE4D dampens glucagon‐driven cAMP signalling, impairs gluconeogenesis and triggers compensatory glycogenolysis.

## 2 Material and Methods

### 2.1 Mice

All animal experiments were approved by the Netherlands Central Authority (CCD No. AVD11800202010784) and followed the Amsterdam UMC Institutional Welfare Committee guidelines. Experiments were mainly conducted in male mice, with mechanistic studies also in females. Control and *Atp8b1* ^*G308V/G308V*^ mutant mice (C57BL/6J) [21] were housed separately in a specific‐pathogen‐free facility on a 12/12 h light/dark cycle, fed standard chow, and given water ad libitum. Mice used were 3 to 4 months old, and all data were scored blinded; sample sizes are noted in figure legends.

### 2.2 Oral Glucose Tolerance Test

Mice were fasted for 5 h and then weighed. Blood glucose samples taken from the tail vein were measured for glucose concentrations pre‐glucose gavage using a Glucometer Contour XT (Bayer). Mice were then orally gavaged with 33% (w/v) D‐glucose (Merck) solution at 2 g/kg, and the post‐gavage glucose levels were measured from the tail vein blood at indicated time points.

### 2.3 Determination of Triglyceride, Free Fatty Acid, and Cholesterol

Liver lipids were extracted according to Srivastava et al. [22]. Plasma triglycerides, non‐esterified free fatty acids (NEFA) and cholesterol were measured using Triglycerides Enzymatic PAP 150 (Trig/GB) kit (Roche), NEFA‐HR (2) reagent (Wako), and Amplex cholesterol assay (ThermoScientific), respectively, according to the manufacturers.

### 2.4 Glycogen Measurement

Glycogen was measured as described before [23]. Briefly, liver lysates were treated with NaOH at 80°C to degrade sugars, cooled, deproteinised with metaphosphoric acid (Sigma) and centrifuged. Samples were incubated with amyloglucosidase (Sigma) to convert glycogen to glucose, then mixed with homo vanillic acid (Sigma) and horseradish peroxidase (Roche). Baseline fluorescence was recorded before adding glucose oxidase (Sigma), and fluorescence was measured at 37°C until completion using a CLARIOstar microplate reader (BMG Labtech).

### 2.5 Isolation, Culture, and Treatment of Primary Mouse Hepatocytes

Primary mouse hepatocytes (PMHs) were isolated as described previously [23]. Hepatocytes were seeded at a density of 100,000 cells/cm^2^ for 3 h in William's medium E (WME; Invitrogen), supplemented with 5% fetal bovine serum (FBS), 100 U/mL penicillin and 100 μg/mL streptomycin (Invitrogen) and 2 mM glutamine (Lonza) in a 5% CO_2_, 37°C incubator after which medium was replaced with FBS‐free WME (WME‐SF). Cells were grown for 24 h or 48 h before treated as described. Stock solution of glucagon (287 μM in 10 mM HCl, pH 2), Rolipram (10 mM in DMSO), Forskolin (10 mM in DMSO) and 3‐isobutyl‐1‐methylxanthine (IBMX, 250 mM in DMSO) were prepared from powder obtained from Sigma‐Aldrich.

### 2.6 Cell Culture and Generation of GCGR Over‐Expression Cells

The human hepatocellular carcinoma cell line HepG2 (ATCC, HB‐8065) and HEK293T cells (ATCC, CRL‐3216) were cultured in high (4.5 g/L) glucose Dulbecco's modified Eagle's medium (DMEM) (Lonza) supplemented with 10% FBS (Lonza), 2 mM L‐glutamine (Lonza), 100 U/mL penicillin (Lonza) and 100 U/mL streptomycin (Lonza) at 37°C in a 10% CO_2_ humidified atmosphere. A 5′‐FLAG‐tagged human GCGR sequence (Genbank NM_000160.5; ordered with Twist Bioscience) was cloned into a third‐generation lentiviral transfer vector (pLV‐CMV‐IRES‐Puro; Cellomics Technology). Lentivirus was produced in HEK293T cells.

### 2.7 Glucagon‐Dependent cAMP Accumulation

Primary hepatocytes, cultured overnight in WME‐SF, were incubated in 250 μL WME‐SF supplemented with 100 nM glucagon with or without 10 μM Rolipram for the time points indicated. cAMP accumulation was terminated after addition of 100 μL lysis buffer (0.35 M HCl/3.5% [w/v] Triton X‐100). Samples were centrifuged for 10 min at 20,000× g at 4°C. The supernatant was harvested, and the cAMP levels were measured using the Direct cAMP ELISA kit (Enzo). cAMP values were normalised to protein.

### 2.8 RNA Isolation, cDNA Synthesis, and Real Time Quantitative Polymerase Chain Reaction (RT‐qPCR)

RNA was isolated from mouse livers, PMH and HepG2 cells using TriPURE reagent (Invitrogen). cDNA was synthesised using random hexamers, oligo‐dT_12‐18_ primer and Superscript‐III RT (Invitrogen). RT‐qPCR was performed on a CFX96 Realtime system C1000 (Bio Rad) using Sensi FAST Sybr Green (Meridian Bioscience). Expression levels were calculated using LinRegPCR software [24] and normalised to reference genes. Human liver RNA was extracted with NucleoZol (Macherey‐Nagel), cDNA synthesised using the High‐Capacity cDNA Reverse Transcription Kit (Applied Biosystems), and qRT‐PCR run with SyGreen Blue Mix (PCR Biosystems) on a Bio‐Rad CFX Duet system. Relative expression was calculated by the ΔΔCT method. Primer sequences are listed in Tables S1 and S2.

### 2.9 SDS‐PAGE, Western Blotting and Surface Biotinylation

Cells were lysed in RIPA buffer supplemented with EDTA‐free protease and phosphatase inhibitors (Roche). Surface biotinylation was performed as described [25]. Protein concentrations were determined by Pierce Bicinchoninic Acid (BCA) protein assay. Lysates were separated by SDS‐PAGE and transferred onto PVDF membranes (Millipore) using a semi‐dry blotting system (Bio‐Rad) with either CAPS/methanol or ethanolamine‐glycine buffer. Membranes were blocked with 5% milk in PBS‐Tween for 1 h at room temperature, then incubated with primary (see Table S3 and [26, 27]) and HRP‐conjugated secondary antibodies diluted in blocking buffer, with washes between incubations. Membranes were developed using enhanced chemiluminescence and imaged on an ImageQuant LAS 4000 (GE Healthcare). Densitometric analysis was performed using ImageJ (http://imagej.nih.gov/ij/).

### 2.10 PFIC1 Patient Cohort and Healthy Control Subjects

Written informed consent was obtained from all patients, and the study conformed to the Declaration of Helsinki with prior approval from institutional human research committees. Liver samples, clinical information, and laboratory data from male and female PFIC1 patients and healthy controls were collected from Amsterdam University Medical Center, Medical University of Innsbruck, National Taiwan University Hospital, and Hannover Medical School under respective IRB approvals. Specifically, paraffin‐embedded liver biopsies from two PFIC1 patients were obtained from Innsbruck (IRB #1029/2017) [28]. Amsterdam UMC provided snap‐frozen and formalin‐fixed paraffin‐embedded (FFPE) liver tissues from two healthy controls (IRB W19_375 #19.440). National Taiwan University Hospital contributed fresh frozen liver samples from four PFIC1 patients [29, 30] and two healthy transplant donors (IRB #200801071R). Hannover Medical School provided FFPE explant liver tissues and clinical data from two PFIC1 patients with informed consent. Clinical and laboratory details for PFIC1 patients and controls are summarised in Tables 1 and 2.

**TABLE 1:** Demographic and clinical summary of PFIC1 patients.

PFIC1 patients	PFIC1 #1	PFIC1 #2	PFIC1 #3	PFIC1 #4	PFIC1 #5	PFIC1 #6	PFIC #7	PFIC #8
Sex	Female	Male	Male	Female	Female	Female	Male	Male
ATP8B1 allele 1	c.181_182ins179	c.1214_1215del	c.2081 T>A (p.Ile694Asn)	c.2707 + 4A>G	deletion of exon7, c.555_627del (p.Phe186LeufsTer27)	c.2821C>T (p.Arg941Ter)	c.2788C>T (p.Arg930Ter)	c.1336G>A (p.Gly446Arg)
ATP8B1 allele 2	c.181_182ins179	c.(2931 + 1_2932–1)_(3400 + 1_3401–1)del	c.2081 T>A (p.Ile694Asn)	c.3554 T>A (p.Leu1185Ter)	c.3391C>T (p.Gln1131Ter)	c.2081 T>A (p.Ile694Asn)	c.2788C>T (p.Arg930Ter)	c.1804C>T (p.Arg602Ter)
Age of first symptoms	2 months	1 month	8 months	1 month	1 month	1 month	3 months	6 months
Age at diagnosis	3 years	2 months	< 1 year	< 1 year	5 years	1.2 years	1.25 years	1.2 years
Age at liver tissue sampling	3 months	21 months	10 months	10 months	5 years	1.7 year	2.7 year	2.8 year
Tissue source	Biopsy	Explant liver	Biopsy	Biopsy	Explant liver	Explant liver	Explanted Liver	Explanted Liver
Snap‐frozen	+	−	+	+	+	+	−	−
FFPE	+	+	−	−	−	−	+	+
Pre‐liver transplantation								
AST (U/L)	94	695	15	54	76	88	91	81
ALT (U/L)	60	402	23	49	94	43	42	52
GGT (U/L)	25	30	14	25	36	24	34	20
Serum bile acids (μmol/L)	320	189	NA	NA	NA	186	187	244
Blood glucose (mg/dL)	81	96	NA	92	NA	NA	92	103
Cholesterol (mg/dL)	151	133	74	185	55	NA	97	143
Triglycerides (mg/dL)	233	196	106	410	134	NA	93	185
Ultrasonography	Hepatosplenomegaly, signs of portal hypertension, rough hepatic surface	Hepatosplenomegaly, signs of portal hypertension, rough hepatic surface	NA	NA	NA	Homogenous increased echogenicity. Surface smooth, margin blunt. No visible intrahepatic lesion.	Signs of portal hypertension and liver fibrosis	Signs of portal hypertension and liver fibrosis
Liver transplantation								
Age of liver transplantation	2.5 years	21 months	NA	NA	5.5 years	1.7 years	2.7 years	2.8 years
Indication of liver transplantation	Acute liver failure, INR 1.5, fibrinogen 87 mg/mL	INR 2.5, fibrinogen 269 mg/mL, pruritus	NA	NA	Liver histologic features showed cirrhosis	INR 1.03, pruritus	Pruritus, grow retardation, liver cirrhosis	Pruritus, grow retardation, liver cirrhosis
Graft	Orthotopic cadaveric liver transplantation	Living donor liver transplantation (father)	NA	NA	Orthotopic cadaveric liver transplantation	Living donor liver transplantation (father)	Orthotopic cadaveric liver transplantation (full size)	Orthotopic cadaveric liver transplantation (SII + III)
Biliary anastomosis	End‐to‐end	Hepaticojejunostomy	NA	NA	Roux‐en‐Y reconstruction with internal stenting (retrocolic)	End‐to‐end	End‐to‐end	End‐to‐end
Post‐liver transplantation								
AST (U/L)	208	326	NA	NA	42	46	36	155
ALT (U/L)	128	262	NA	NA	16	40	26	149
GGT (U/L)	27	72	NA	NA	292	57	5	286
Blood glucose (mg/dL)	78	98	NA	NA		NA	104	40
Cholesterol (mg/dL)	76	101	NA	NA	88	NA	104	120
Triglycerides (mg/dL)	68	71	NA	NA	85	NA	NA	112
Liver biopsy	Steatosis (> 90%) with focal and mild steatohepatitis.	Mild to moderate portal tract fibrosis, mild lobular chronic inflammation, steatosis (20%).	NA	NA	Expanded portal area with focal incomplete septum formation. Scanty inflammatory cell infiltration with moderate bile duct damage. Minimal endothelial damage. Moderate acute rejection is considered.	Moderate steatosis. mixed macrovesicular and microvesicular steatosis. Mild and focal ductular reaction.	NA	40% Steatosis, advanced Fibrosis ISHAK F4‐5 (8 years. after liver transplantation)
Diarrhoea (per day)	4–7	10–12	NA	NA	NA	3	5–10	3–4
Other remark	NA	NA	NA	NA	Acute rejection	NA	Steatosis, colitis, IBATi treatment	Steatosis, IBATi treatment

Abbreviations: ALT, alanine transaminase; AST, aspartate transaminase; FFPE, formalin‐fixed paraffin‐embedded; GGT, γ‐glutamyltransferase; IBATi, ileal bile acid transporter inhibitor; NA, not available.

**TABLE 2:** Demographic and clinical summary of healthy control subjects.

Healthy controls	HC #1	HC #2	HC #3	HC #4
Sex	Male	Male	Female	Female
Age	30–40	40–50	40–50	30–40
Tissue source	Surgical resection	Surgical resection	Unused donor liver	Unused donor liver
Snap‐frozen	+	+	+	+
FFPE	+	+	−	−

### 2.11 Neutral Lipid Staining

Oil Red O staining was performed on 6 μm liver cryosections fixed in 3.7% (w/v) formaldehyde for 1 h at room temperature. Sections were washed, stained with Oil Red O (Sigma) working solution for 30 min, rinsed in running tap water for 10 min, mounted in glycerol‐gelatin and imaged using an Olympus BX51 microscope.

### 2.12 Immunohistochemistry

Five‐micron sections were deparaffinised, rehydrated and endogenous peroxidase quenched with 0.3% H_2_O_2_ in methanol. After antigen retrieval at 120°C in sodium citrate buffer, slides were blocked with 1% BSA/PBS and incubated overnight at 4°C with primary antibodies against PDE4D or p‐CREB. Binding was detected using HRP‐labelled secondary antibodies (Immunologic) and diaminobenzidine (Sigma‐Aldrich), followed by Mayer's haematoxylin counterstaining. Sections were imaged in an Olympus BX51 microscope. PDE4D‐positive surface area quantification was performed blinded on at least 8 non‐overlapping sections of 2 PFIC1/healthy controls (HCs) transplant livers and 2 *Atp8b1*/WT mutant livers by applying intensity thresholds to grayscale images in ImageJ 1.50i (http://imagej.nih.gov/ij/), generating binary masks that isolate positive staining. Area and mean intensity metrics within the thresholded regions were quantified to assess staining levels. Threshold values were manually optimised to minimise background inclusion.

### 2.13 Periodic Acid‐Schiff (PAS) Staining and PAS‐Diastase (PAS‐D) Staining

PAS‐D staining was performed on FFPE liver sections to detect glycogen. Sections were deparaffinised, rehydrated and incubated with or without 2.5 mg/mL α‐amylase (Sigma) for 30 min at room temperature. After washing, slides were treated with 1% periodic acid (Sigma) for 8 min, washed again and stained with Schiff's reagent (Merck) for 10 min. Following a tap water wash, sections were dehydrated, mounted in Entellan (Merck) and imaged using an Olympus BX51 microscope.

## 3 Results

### 3.1 Deficiency of ATP8B1 in Mice Causes Fasting Hypolipidaemia, Hypoglycaemia, and Hepatic Steatosis

**FIGURE 1:**
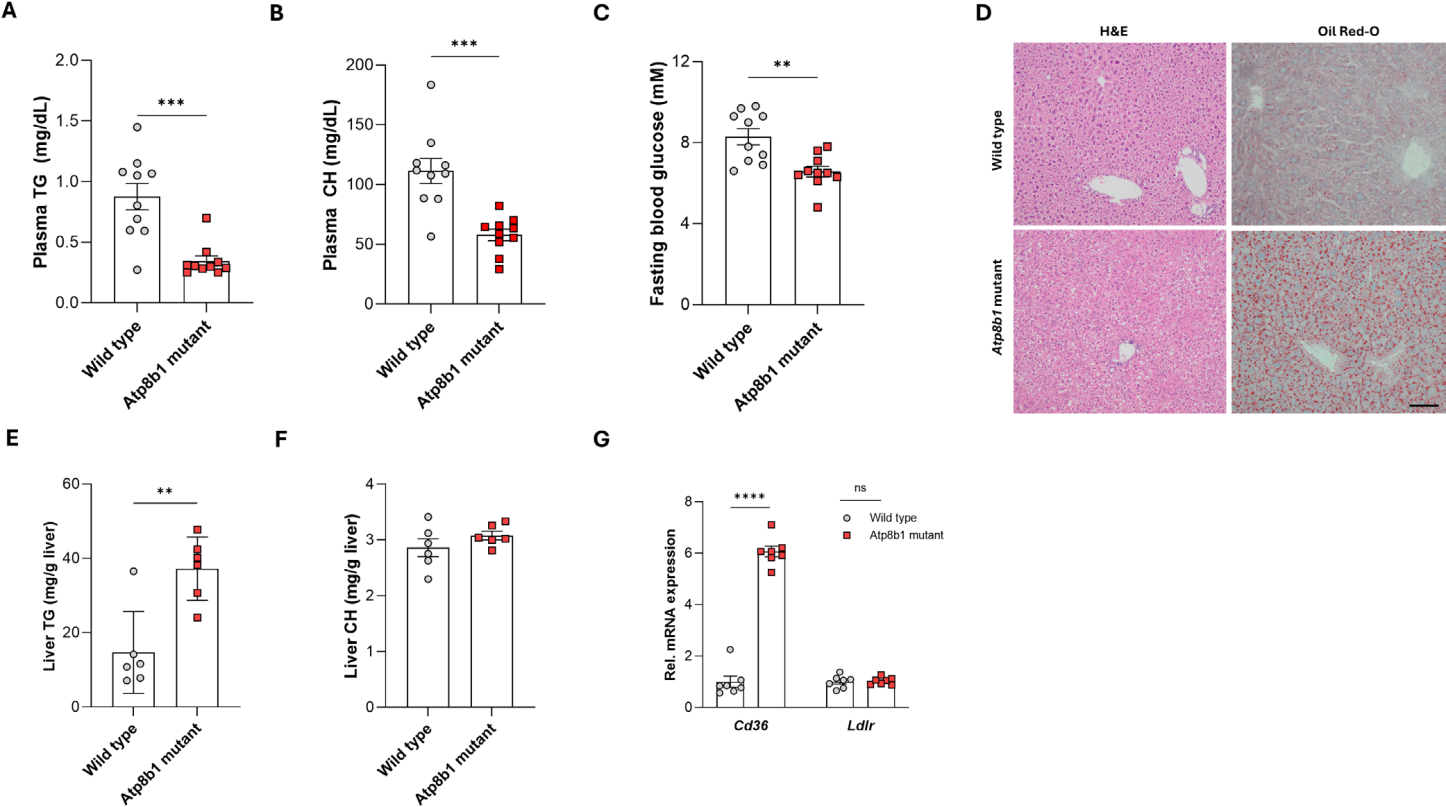
Fasting hypolipidaemia and hypoglycaemia and hepatic steatosis in *Atp8b1* mutant mice. Systemic and hepatic metabolic parameters in 3‐month‐old male C57BL/6J wild type and *Atp8b1* mutant mice fed a regular chow diet (*n* = 10 for A‐C; *n* = 6 for E, F; *n* = 7 for G). (A) Plasma triglyceride (TG) levels. (B) Plasma cholesterol (CH) levels. (C) Fasting blood glucose levels. (D) Haematoxylin–eosin (H&E)‐ and Oil Red‐O staining shows neutral lipid accumulation in *Atp8b1* mutant liver. Scale bar = 100 μm. (E) Hepatic triglyceride (TG) and (F) cholesterol levels (CH). (G) *Cd36* and *Ldlr* mRNA expression in liver of *Atp8b1* mutant mice. Statistical analysis by Student's *t*‐test; **p < 0.005; ***p < 0.0005; ****p < 0.0001.

To understand the metabolic consequence of ATP8B1 deficiency, we investigated the systemic metabolic profile in *Atp8b1* ^*G308V/G308V*^ mice (hereafter *Atp8b1* mutant mice). In contrast to hyperlipidaemia as observed in cholestatic PFIC1 patients [10, 11], *Atp8b1* mutant mice (without cholestasis) did not show signs of hyperlipidaemia, as evidenced by reduced fasting plasma levels of triglyceride and cholesterol (Figure 1A,B). Compared to wild type (WT) controls, *Atp8b1* mutant mice had reduced fasting blood glucose levels (Figure 1C). Liver histology and Oil Red O staining showed accumulation of neutral lipids in *Atp8b1* mutant livers (Figure 1D). Enzymatic quantification of hepatic lipids revealed increased triglyceride levels but unaffected total cholesterol levels in *Atp8b1* mutant livers (Figure 1E,F). Interestingly, was this phenotype associated with six‐fold higher expression of the fatty acid transporter *Cd36*, but not of the low‐density lipoprotein receptor *Ldlr* (Figure 1G). These data show that, in the absence of cholestasis, ATP8B1 deficiency causes hypolipidaemia, hepatic steatosis and fasting hypoglycaemia. Because intestinal ATP8B1 deficiency is known to cause hepatic steatosis [9], we focused on the effect of ATP8B1 deficiency on glucose metabolism.

### 3.2 *Atp8b1* Mutant Mice Are Glucose‐Tolerant but Have Impaired Gluconeogenesis

**FIGURE 2:**
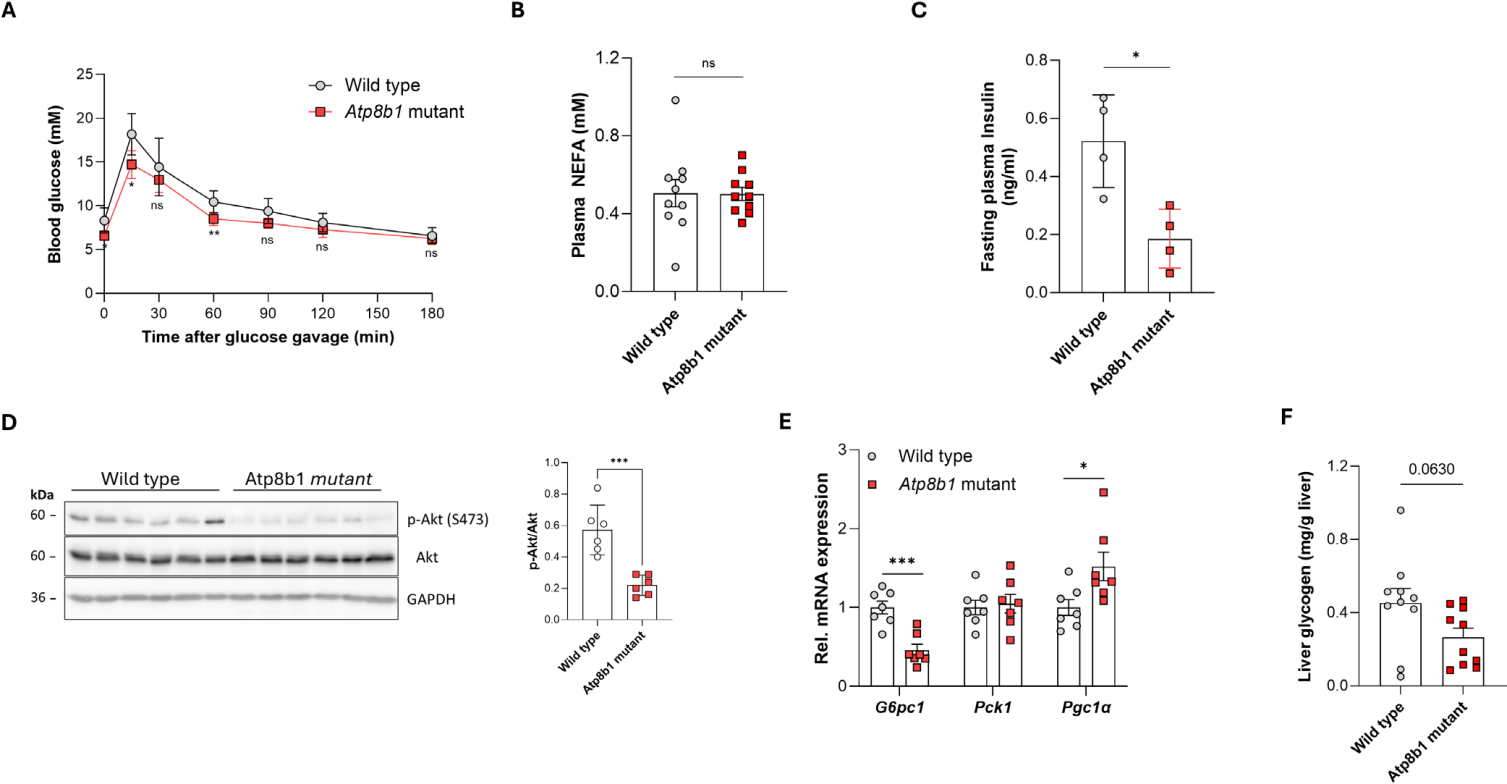
*Atp8b1* mutant mice are glucose tolerant and display signs of impaired gluconeogenesis. (A) Oral glucose tolerance test shows no difference in glucose handling between male wild type and *Atp8b1* mutant mice; *n* = 6 mice per genotype. (B) Fasting plasma non‐esterified fatty acids (NEFA; *n* = 10). (C) Fasting plasma insulin levels (*n* = 4). (D) Akt and p‐Akt protein levels in freshly isolated hepatocytes directly snap‐frozen after isolation from fasted wild type and *Atp8b1* mutant livers. Densitometric analysis of *n* = 6 technical replicates. Statistical significance with Student's *t*‐test; ***p < 0.0005. (E) Hepatic expression of genes involved in gluconeogenesis. mRNA expression was corrected for the geometric mean of *36b4*, *ß‐actin*, *Tbp* and *Hprt* mRNA, normalised to wild type and expressed as mean ± standard deviation (*n* = 7). (F) Hepatic glycogen levels (*n* = 10). All statistical analyses by Student's *t*‐test; *p < 0.05; **p < 0.05; ***p < 0.0005.

Because blood glucose levels are regulated by insulin and glucagon signalling, we examined the insulin sensitivity in *Atp8b1* mutant mice. Oral glucose tolerance testing indicated no difference in insulin sensitivity between WT and *Atp8b1* mutant mice, but a reduced baseline glucose level in *Atp8b1* mutant mice (Figure 2A). In addition, the fasting plasma non‐esterified fatty acid levels were normal in the *Atp8b1* mutant mice, also indicating normal insulin sensitivity in the peripheral tissues (Figure 2B). Consistently, fasting plasma insulin levels were reduced in *Atp8b1* mutant mice (Figure 2C), while Akt signalling was ~50% reduced in freshly isolated *Atp8b1* mutant hepatocytes (Figure 2D), ruling out hyperinsulinaemia and insulin hypersensitivity as the cause of reduced fasting glucose and suggesting that *Atp8b1* mutant mice have impaired gluconeogenesis. Indeed, compared to WT mice, *Atp8b1* mutant mice had 55% reduced hepatic expression of glucose‐6‐phosphatase (*G6pc1*) despite a slightly upregulation of peroxisome proliferator‐activated receptor gamma coactivator 1‐alpha (*Pgc1a*) (Figure 2E). The expression of phosphoenolpyruvate carboxykinase (*Pck1*) was not affected in *Atp8b1* mutant mice. In addition to hypoglycaemia and reduced hepatic *G6pc1* expression, hepatic glycogen stores, an additional source for hepatic glucose output, tended to be reduced in fasted *Atp8b1* mutant mice compared to WT mice (Figure 2F). Together, the data suggest that *Atp8b1* mutant mice have normal insulin sensitivity but have an impaired glucagon response.

### 3.3 Glucagon‐Dependent cAMP Signalling Is Impaired in *Atp8b1* Mutant Hepatocytes

**FIGURE 3:**
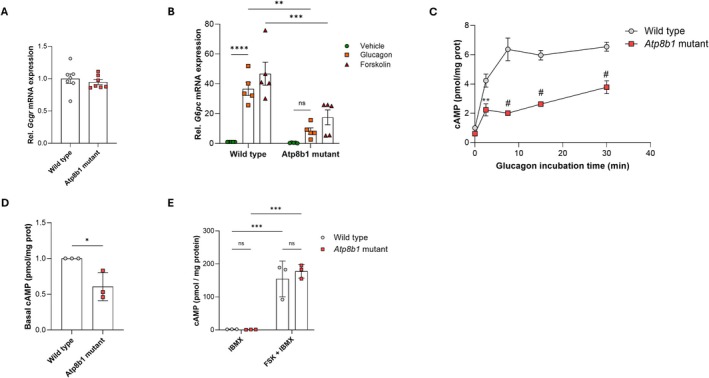
Glucagon‐dependent cAMP signalling is impaired in *Atp8b1* mutant hepatocytes. (A) Glucagon receptor (*Gcgr*) expression in mouse liver (*n* = 7). (B) *G6pc1* mRNA expression in male wild type and *Atp8b1* mutant primary hepatocytes incubated for 3 h with 100 nM Glucagon or 10 μM Forskolin. mRNA expression was corrected for the geometric mean of *ß‐Actin* and *Hprt* mRNA, normalised to wild type—Vehicle and expressed as mean ± standard deviation of *n* = 5 independent hepatocyte isolations. Statistical analyses by two‐way ANOVA with Tukey correction for multiple analyses; **p < 0.005; ***p < 0.001; ****p < 0.0001. (C) cAMP accumulation is strongly reduced in male *Atp8b1* mutant hepatocytes. Hepatocytes were incubated with 100 nM Glucagon for the indicated time points and cAMP was measured as described in Section 2. Data were normalised to wild type (*t* = 0 min) and are expressed as mean ± standard error of the mean of *n* = 3 independent hepatocyte isolations. Statistical analyses by Student's *t*‐test; **p < 0.005; ^#^ *p* < 0.0001. (D) Basal cAMP levels of freshly isolated hepatocytes. Data were normalised to wild type levels and expressed as mean ± standard deviation of *n* = 3 independent isolations. Statistical analyses by Student's *t*‐test; *p < 0.05. (E) cAMP levels after 5 min 10 μM Forskolin (FSK)/250 μM IBMX incubation. Data were normalised to wild type and expressed as mean ± standard deviation of *n* = 3 independent isolations. Statistical analyses by two‐way ANOVA with Tukey correction for multiple analyses; ***p < 0.001.

We next investigated how ATP8B1 deficiency leads to impaired induction of gluconeogenesis genes using PMHs. Although the expression of GCGR was not affected in *Atp8b1* mutant livers (Figure 3A), stimulating *Atp8b1* mutant PMHs with glucagon failed to induce the expression of *G6pc1* (Figure 3B), indicating that glucagon‐dependent cAMP signalling was impaired. Stimulation with forskolin, a pan‐adenylyl cyclase activator, also failed to restore *G6pc1* expression in *Atp8b1* mutant PMHs. Indeed, glucagon‐treated *Atp8b1* mutant PMHs accumulated significantly less cAMP in comparison to WT PMHs (Figure 3C). Notably, even in the absence of glucagon, basal cAMP levels in *Atp8b1* mutant PMHs were significantly lower than those of WT PMHs (Figure 3D), suggesting that there was either decreased cAMP production by adenylyl cyclases or increased cAMP degradation by PDEs. To test if the reduced cAMP level in glucagon‐stimulated *Atp8b1* mutant PMHs was caused by reduced adenylyl cyclase activity, we quantified cAMP levels in the presence of forskolin and the broad‐spectrum PDE inhibitor, IBMX. *Atp8b1* mutant and WT PMHs produced comparable amounts of cAMP following treatment with IBMX and forskolin (Figure 3E), indicating that not cAMP production, but increased cAMP degradation impaired glucagon signalling in *Atp8b1* mutant PMHs.

### 3.4 Upregulation of PDE4 Mediates Glucagon Resistance in *Atp8b1* Mutant Hepatocytes

**FIGURE 4:**
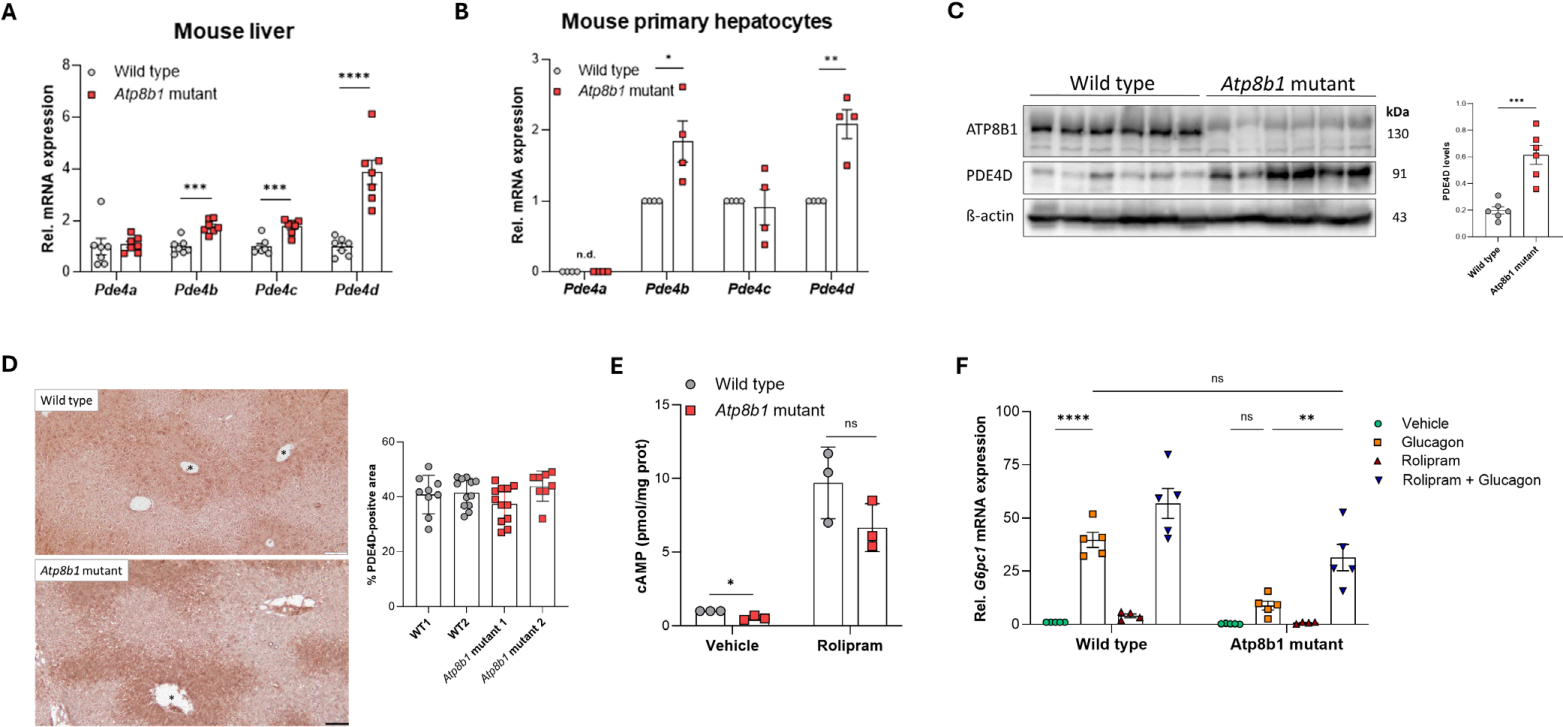
Upregulation of phosphodiesterase 4 (PDE4) causes glucagon resistance in *Atp8b1* mutant hepatocytes. (A) mRNA expression of *Pde4* isoforms in male mouse liver. mRNA expression was corrected for the geometric mean of *36b4*, *ß‐Actin*, *Tbp* and *Hprt* mRNA. (B) mRNA expression of *Pde4* isoforms in cultured primary male mouse hepatocytes. mRNA expression was corrected for the geometric mean of *ß‐Actin* and *Hprt* mRNA; n.d. = not detectable. Data in (A) and (B) were normalised to wild type and expressed as mean ± standard error of the mean of *n* = 7 mice (A) and mean ± standard deviation of *n* = 4 independent hepatocyte isolations (B). (C) ATP8B1 and PDE4D protein levels in mouse liver (*n* = 6). Quantification of PDE4D by densitometry and normalisation for ß‐Actin. (D) Immunohistochemical detection of PDE4D in mouse liver. Bar = 100 μm. Asterisk indicates the central vein. PDE4D immune‐positive surface area was quantified as described in material and methods. Every data point represents the percentage of PDE4D‐positivity of a non‐overlapping tissue section. (E) cAMP levels of primary hepatocytes incubated for 15 min with 100 nM glucagon in the presence or absence of 10 μM Rolipram. Data were normalised to wild type vehicle levels and expressed as mean ± standard deviation of *n* = 3 independent isolations. (F) *G6pc1* mRNA expression in male wild type and *Atp8b1* mutant primary mouse hepatocytes incubated for 3 h with 100 nM Glucagon, 10 μM Rolipram or Glucagon/Rolipram. mRNA expression was corrected for the geometric mean of *ß‐Actin* and *Hprt* mRNA, normalised to wild type—vehicle and expressed as mean ± standard deviation of *n* = 4–5 independent hepatocyte isolations. Statistical analyses by two‐way ANOVA with Tukey correction for multiple analyses; **p < 0.005; ****p < 0.0001. All other statistical analyses by Student's *t*‐test; *p < 0.05; **p < 0.005; ***p < 0.0005; ****p < 0.0001.

Because the cAMP‐degrading phosphodiesterase PDE4 isoforms are known to regulate hepatic glycogenolysis and gluconeogenesis [17], we assessed the expression of *Pde4* isoforms in whole liver and found *Pde4b* and *Pde4c* mildly increased (~twofold), while *Pde4d* expression was increased fourfold in male *Atp8b1* mutant livers (Figure 4A). Importantly, all other *Pde* isoforms known to be expressed in mouse hepatocytes (www.livercellatlas.org), except for *Pde8a*, which was mildly down‐regulated, were not affected in *Atp8b1* mutant liver (Figure S1A). *Pde4d* expression was not affected in the colon of *Atp8b1* mutant mice (Figure S1B). *Pde4b* and *Pde4d* were increased ~twofold in overnight‐cultured *Atp8b1* mutant PMHs (Figure 4B). Immunoblotting of whole liver lysates showed a threefold increase in PDE4D protein levels in *Atp8b1* mutant livers (Figure 4C). Immunohistochemistry revealed that PDE4D was localised to mid‐zone and pericentral hepatocytes in the liver of WT and *Atp8b1* mutant liver, while this distribution was not affected in *Atp8b1* mutant liver (Figure 4D). Importantly, Rolipram, a PDE4‐selective inhibitor, largely restored cAMP production as well as induction of *G6pc1* expression in glucagon‐stimulated male *Atp8b1* mutant PMHs (Figure 4E,F).

Similar to male mice, female *Atp8b1* mutant liver also showed a ~twofold reduced expression of *G6pc1* while only a mild (~twofold, as opposed to fourfold in males) upregulation of *Pde4d* was observed (Figure S1C). Although female PMHs were mildly glucagon insensitive, this could not be rescued by rolipram (Figure S1D). Overall, our results show that upregulation of PDE4D in male *Atp8b1* mutant PMHs impairs glucagon‐dependent cAMP signalling and transcriptional induction of *G6pc1*, which could be rescued by PDE4‐selective inhibition.

### 3.5 PFIC1 Patients Have Increased Hepatic *PDE4* Expression and Reduced Glycogen Content

**FIGURE 5:**
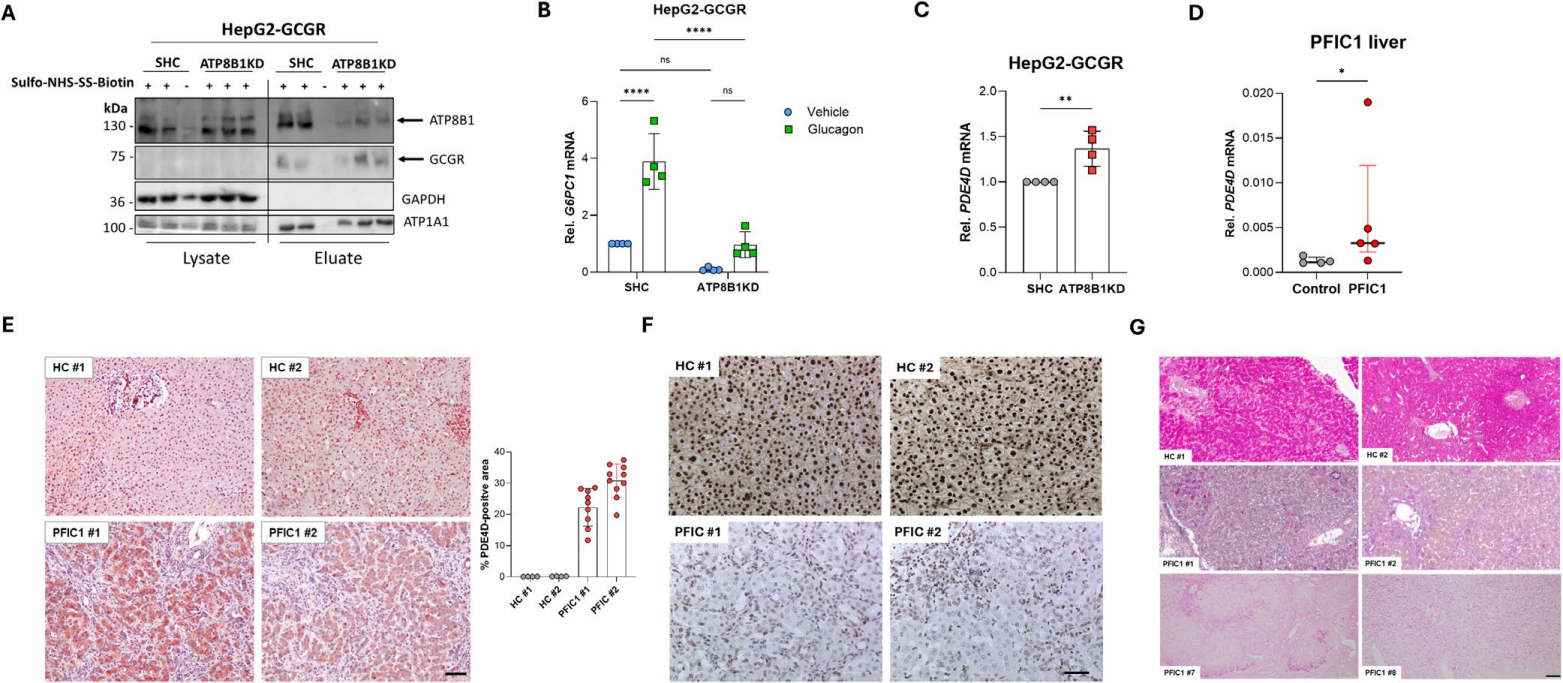
PDE4D Is increased in ATP8B1 KD HepG2‐GCGR cells as well as in PFIC1 patient liver. (A) Cell surface biotinylation of ATP8B1KD HepG2‐GCGR cells. ATP8B1 and GCGR in the lysate fractions were below the detection limit. In the eluate (plasma membrane) fraction, the ATP8B1 level was strongly reduced in ATP8B1KD HepG2, while the GCGR level was not affected by ATP8B1 knockdown. (B) *G6PC1* expression in HepG2‐GCGR cells shows a significant reduction in glucagon‐mediated signalling in ATP8B1 KD cells. (C) *PDE4D* expression in HepG2‐GCGR cells. (D) *PDE4D* expression in pre‐transplant liver of PFIC1 patients (*n* = 4–5, see Table 1). Expression was corrected for the geometric mean of *36B4* and *ß‐Actin* and expressed as mean ± standard deviation. Statistical analyses by Mann–Whitney test; *p < 0.05. (E) Immunohistochemical staining of PDE4D in pre‐transplant liver of two PFIC1 patients. PDE4D immune‐positive surface area was quantified as described in material and methods. Every data point represents the percentage of PDE4D‐positivity of a non‐overlapping tissue section. Bar = 100 μm. (F) Immunohistochemical staining of p‐CREB in pre‐transplant liver of PFIC1 patients. Bar = 50 μm. (G) Glycogen detection in liver sections of two controls and four PFIC1 patients using PAS staining. Serial sections were pretreated the glycogen‐degrading enzyme amylase (PAS‐D) after which the sections were stained with PAS (see Figure S3B). The magenta colour, which after pretreatment of the sections with amylase completely disappears, represents glycogen. Hardly any glycogen staining is detected in PFIC1 livers. Bar = 100 μm. Data in (B) and (C) were normalised to HepG2‐SHC, and are expressed as mean ± standard deviation of 4 independent experiments. Statistical analysis in (B) by two‐way ANOVA with Tukey correction for multiple testing; ****p < 0.001. Statistical analysis in (C) by Student's *t*‐test; **p < 0.005.

Finally, we examined if ATP8B1 deficiency also impaired glucagon signalling in HepG2 cells and human PFIC1 livers. Because parental HepG2 cells did not express GCGRs and did not upregulate *G6PC1* in response to glucagon (Figure S2A), we overexpressed the human GCGR in HepG2 cells (hereafter HepG2‐GCGR cells; Figure S2B). In contrast to HepG2 parental cells, HepG2‐GCGR cells were responsive to glucagon as evidenced by the induction of *G6PC1* expression (Figure S2C). To model ATP8B1 deficiency in human hepatocytes, we knocked down ATP8B1 in HepG2‐GCGR cells. ATP8B1 knockdown did not affect GCGR surface expression as shown by surface biotinylation (Figure 5A). However, similar to *Atp8b1* mutant PMHs, ATP8B1 knockdown in HepG2‐GCGR cells resulted in impaired glucagon‐dependent *G6PC1* expression (Figure 5B) and a concomitant up‐regulation of *PDE4D* expression (Figure 5C). To translate our findings in *Atp8b1* mutant mice, *Atp8b1* mutant PMHs and ATP8B1 knockdown HepG2‐GCGR cells, we examined the mRNA and protein expression of PDE4D in the native livers of PFIC1 patients (Table 1) and healthy control subjects (Table 2). *PDE4D* was significantly upregulated in PFIC1 livers compared to healthy control livers (Figure 5D). In contrast to *Atp8b1* mutant liver, PFIC1 liver showed neither steatosis nor elevated *CD36* or *LDLR* expression (Figure S3A). Notably, immunohistochemistry revealed clearly increased PDE4D immunoreactivity in hepatocytes of PFIC1 liver, while this was not detected in the liver of HC (Figure 5E). Importantly, immunohistochemical detection of phospho‐CREB showed a strongly reduced nuclear staining in hepatocytes of (fasted) PFIC1 liver, which is consistent with a defective cAMP‐PKA‐CREB signalling cascade due to PDE4D upregulation (Figure 5F). Moreover, PAS staining and PAS‐D staining revealed a near complete depletion of hepatic glycogen in PFIC1 livers (Figure 5G, Figure S3B), which indicates that PFIC1 livers had enhanced glycogenolysis to compensate for impaired gluconeogenesis and to maintain a normal blood glucose level.

## 4 Discussion

Under hypoglycaemic conditions, glucagon promotes hepatic gluconeogenesis and hepatic glucose output by GCGR‐dependent cAMP signalling [12]. The cAMP pool generated by GCGR‐dependent activation of adenylyl cyclase is compartmentalised, with the cAMP concentration gradient controlled by the balance between the plasma membrane adenylyl cyclases and the cAMP‐degrading PDEs. In the present study, we show that ATP8B1 deficiency in mice leads to glucagon resistance and fasting hypoglycaemia and identify that increased PDE4 expression in human and mouse ATP8B1‐deficient hepatocytes mediates this glucagon resistance. First, *Atp8b1* mutant hepatocytes were insensitive to glucagon‐dependent induction of gluconeogenic (*G6pc1*) gene expression, and ATP8B1 knockdown in HepG2‐GCGR cells abolished glucagon‐dependent *G6PC1* induction. Second, compared to WT PMHs, *Atp8b1* mutant PMHs had significantly lower cAMP levels both at baseline and after glucagon stimulation, indicating enhanced cAMP degradation by PDEs. Third, *Atp8b1* mutant mice have increased expression of *Pde4b, Pde4c* and *Pde4d* in the liver. Fourth, rolipram‐mediated inhibition of PDE4 activity restored cAMP levels as well as glucagon‐dependent *G6pc1* mRNA expression in male *Atp8b1* mutant PMHs. Finally, native livers of PFIC1 patients showed markedly increased PDE4D and strongly reduced nuclear p‐CREB staining in hepatocytes.

Although further experiments are required to conclusively establish PDE4D's causative role in vivo, the increased PDE4D and the reduced nuclear p‐CREB levels in PFIC1 liver strongly suggest that PFIC1 patients, like the *Atp8b1* mutant mice, have a defect in glucagon‐dependent cAMP signalling, leading to impaired gluconeogenesis. Despite the lack of direct evidence of glucagon resistance in PFIC1 patients, the near absence of glycogen in native PFIC1 livers suggests that the PFIC1 liver compensates impaired hepatic gluconeogenesis with enhanced glycogenolysis to maintain normal blood glucose levels during fasting. Although cirrhosis of PFIC1 livers could contribute to decreased hepatic glycogen levels, a trend of enhanced compensatory glycogenolysis was also observed in the (non‐cirrhotic) livers of *Atp8b1* mutant mice. In addition to hepatic glucagon resistance, increased PDE4 expression in hepatocytes can be expected to dampen cAMP signalling of other G‐protein‐coupled receptors, such as β_2_‐adrenergic receptor signalling, which can mobilise lipases adipose triglyceride lipase (ATGL) and hormone‐sensitive lipase (HSL) to promote lipolysis of lipid droplets [31].

We show that *Atp8b1* mutant mice were glucose tolerant and, surprisingly, had reduced plasma insulin levels. PDE4‐mediated hepatic glucagon resistance and fasting hypoglycaemia likely contribute to the suppression of insulin secretion by β‐cells in the pancreas. In addition, Ansari and colleagues previously showed that ATP8B1 knockdown in a human pancreatic beta cell line as well as in rat and human pancreatic islands resulted in a ~50% reduction in glucose‐induced insulin release from these cells [32], which may explain the reduced insulin levels in *Atp8b1* mutant mice. Insulin is known to suppress hepatic secretion of Insulin‐like growth factor‐binding protein 1 (IGFBP‐1), a plasma protein that binds and inhibits insulin‐like growth factor 1 (IGF‐1) signalling [33]. Because IGF‐1 is the key effector of growth hormones and inhibits hair cell apoptosis [34], we speculate that chronic suppression of IGF‐1 signalling due to reduced blood insulin levels can contribute to growth failure and hearing impairment in PFIC1 patients [5] and *Atp8b1* mutant mice [6, 21].

In the present study, we observed hepatic steatosis in non‐cholestatic, male *Atp8b1* whole body mutant mice, which highly resembles graft steatosis in PFIC1 patients after liver transplantation [7, 8, 35, 36]. The steatosis of graft livers in PFIC1 patients is presumably caused by extra‐hepatic ATP8B1 deficiency. This assumption is strengthened by recent observations by Tamura and colleagues [9], who showed that knockout of ATP8B1 in intestinal epithelial cells in mice (*Atp8b1* ^*IEC‐KO*^) caused hepatic steatohepatitis. The authors showed that male *Atp8b1* ^*IEC‐KO*^ mice as well as pre‐ and post‐transplant PFIC1 patients had reduced plasma choline levels [9]. Choline supplementation of the diet rescued the steatosis phenotype in male *Atp8b1* ^*IEC‐KO*^ mice, suggesting that impaired intestinal choline absorption causes steatohepatitis in mice and post‐transplant steatosis in the PFIC1 patients [9]. Hence, the hepatic steatosis observed in our mice may thus be caused by intestinal deficiency of ATP8B1. Still, we cannot exclude that increased hepatic PDE4D expression and impaired glucagon‐cAMP signalling contribute to hepatic steatosis in *Atp8b1* mutant mice. First, glucagon is known to stimulate ß‐oxidation of fatty acids in mice and in healthy humans as well as in subjects with MASLD/MASH [37, 38]. Second, a high‐fat diet induces hepatic steatosis with a concomitant increase of hepatic PDE4D in mice, while over‐expression of PDE4D by adeno‐associated virus 8 in wild‐type livers directly resulted in hepatic steatosis in the absence of a high‐fat diet [19]. Notably, PDE4D overexpression in mouse liver increased CD36 expression [19], suggesting that the PDE4D‐CD36 axis might contribute to the hepatic steatosis observed in *Atp8b1* mutant mice. Finally, hepatic steatosis in ALD coincided with reduced cAMP levels and increased PDE4A‐PDE4D isoform levels, both in alcohol‐fed mice and patients with ALD; Rolipram‐mediated PDE4 inhibition restored cAMP levels in the liver of these mice and attenuated lipid accumulation, underscoring the importance of regulated PDE4‐mediated cAMP signalling in lipid metabolism [20, 39].

An overt difference between *Atp8b1* mutant mice and PFIC1 patients is hepatic steatosis, which is not observed in pre‐transplant PFIC1 patients but is strikingly prevalent in post‐transplant PFIC1 patients [7, 8, 35, 36]. Several explanations on top of impaired intestinal absorption of choline (see above) are possible. First, in contrast to pre‐transplant PFIC1 patients, which present with strongly elevated plasma bile salt levels and hepatic bile salt retention, *Atp8b1* mutant mice have only marginally elevated plasma bile salt levels and an unaffected bile flow and bile salt secretion [21]. Because graft steatosis in post‐transplant PFIC1 patients can be prevented or reversed by internal biliary diversion effectively [40], the (unaffected) enterohepatic cycling of bile salts in the *Atp8b1* mutant mice likely contributes to the development of hepatic steatosis. Second, hepatic steatosis in *Atp8b1* mutant liver coincided with increased expression of *Cd36*, which is involved in the uptake of medium‐ and long‐chain fatty acids. In pre‐transplant PFIC1 liver, on the other hand, *CD36* expression was not induced, which is consistent with the absence of steatosis. It will be of interest to assess *CD36* expression in post‐transplant (steatotic) PFIC1 livers. Finally, hepatic bile salt retention could, despite causing liver pathology, engage metabolic or anti‐inflammatory transcriptional programmes to reverse steatosis for instance via FXR activation [41]. Indeed, *Fxr* ^*−/−*^ mice develop hepatic steatosis [42], whereas FXR activation by bile salts reduces hepatic fat accumulation [43].

Our data suggest a causal link between ATP8B1 and PDE4D. PDE4D is upregulated in livers of *Atp8b1* mutant mice and PFIC1 patients and increases following ATP8B1 knockdown in HepG2‐GCGR cells. An inverse correlation between hepatic *Pde4d* and *Atp8b1* expression was also observed across 41 inbred mouse strains [44] (Figure S4); hepatic transcriptomic data deposited by Wu et al. [45], suggesting ATP8B1 fine‐tunes hepatic PDE4D levels independently of genetic or dietary interventions. This regulation is tissue‐specific, as colon *Pde4d* expression remained unchanged in mutants. While further experiments are needed to establish a direct causal link between ATP8B1 and PDE4D, we can propose a potential mechanism connecting both proteins, possibly involving ATP8B1's function as a phospholipid flippase [2]. ATP8B1 promotes local clustering of phosphatidylserine to create docking sites for small GTPases [46], regulating their activity. A similar process might regulate *PDE4D* expression; the stability of *PDE4D* mRNA is negatively regulated by a protein kinase C (PKC) signalling pathway [47]. Since PKC activation depends on phosphatidylserine interaction, ATP8B1 deficiency could impair PKC signalling, leading to increased *PDE4D* expression.

PDE4D activity is also regulated by PKA‐dependent phosphorylation within cAMP signalling microdomains [48], which involves several phospholipid‐interacting scaffolding proteins, potentially linking it to ATP8B1. For instance, the scaffolding protein coiled‐coil and C2 domain containing 1A (CC2D1A) has a high affinity for phosphatic acid and phosphatidylserine and modulates cAMP signalling [49]. Interestingly, mutation of *Cc2d1a* in the hippocampus of mice leads to male‐selective PDE4D hyperactivity, which was reversed by PDE4D inhibition [50]. The latter example highlights potential sex‐specific regulation of cAMP signalling by PDE4D in *Atp8b1* mutant hepatocytes, as female hepatocytes exhibited less *Pde4d* upregulation and no Rolipram‐induced rescue of glucagon signalling, suggesting sex‐biased mechanisms. Further study is needed to determine if ATP8B1 regulates PDE4D activity, transcriptionally and post‐translationally, by controlling local membrane phospholipid distribution.

Our study uncovers a novel role of hepatic ATP8B1 in regulating glucose metabolism. ATP8B1 deficiency in the liver leads to PDE4D‐mediated suppression of cAMP signalling, causing glucagon resistance, impaired gluconeogenesis, and compensatory glycogenolysis in hepatocytes. These findings identify PDE4D as a potential therapeutic target for liver pathology in PFIC1 patients and provide new metabolic insights into the mechanisms underlying their extrahepatic anomalies.

## Author Contributions

**Jung‐Chin Chang:** conceptualisation, methodology, investigation, formal analysis, writing – original draft, writing – review and editing, visualisation. **Wietse In het Panhuis:** investigation, writing – review and editing. **Shu‐Hao Hsu:** investigation, resources, visualisation, writing – review and editing. **Suzanne Duijst:** investigation, visualisation, validation. **Kam S. Ho‐Mok:** investigation, visualisation, validation. **Jolie F. van der Heiden:** investigation, visualisation, writing – review and editing. **Zhixiong Ying:** investigation, writing – review and editing. **Joris I. Erdmann:** investigation, resources. **Georg F. Vogel:** investigation, resources, writing – review and editing. **Norman Junge:** investigation, resources, writing – review and editing. **Björn Hartleben:** investigation, resources. **Pim J. Koelink:** investigation, visualisation. **Sebastian G. van Dijk:** investigation, visualisation, writing – review and editing. **Weng Chuan Peng:** investigation, visualisation. **Arthur J. Verhoeven:** investigation, writing – review and Editing. **Ronald P. J. Oude Elferink:** investigation, writing – review and editing. **Huey‐Ling Chen:** investigation, resources. **Stan F. J. van de Graaf:** investigation, writing – review and editing. **Coen C. Paulusma:** conceptualiaation, methodology, investigation, formal analysis, writing – original draft, writing – review and editing, visualisation, supervision, project administration.

## Conflicts of Interest

Georg F. Vogel consults for and receives scientific grants from Mirum Pharmaceuticals, Albireo/Ipsen Pharma, and Takeda Pharmaceuticals. Stan F. J. van de Graaf consults for ProQR Therapeutics.

## Supporting information


**Tables S1**–**S3:** liv70306‐sup‐0001‐TableS1‐S3.docx.

**Figures S1**–**S4:** liv70306‐sup‐0002‐FigureS1‐S4.docx.

## Data Availability

The data that support the findings of this study are available from the corresponding author upon reasonable request.
