# Supplementary material for: ATP8B1 Deficiency Causes Phosphodiesterase 4‐Mediated Glucagon Resistance and Impaired Gluconeogenesis in Mouse and Human Liver
Source: Liver Int. 2025 Aug 25;45(9):e70306. doi: 10.1111/liv.70306 (PMC12375944; doi:10.1111/liv.70306)
Supplement: Supplementary file 1 — Tables S1–S3: liv70306‐sup‐0001‐TableS1‐S3.docx. [file LIV-45-0-s001.docx]

**Supplementary Table 1** Mouse oligonucleotide sequences used in this study.

| Gene | Forward (5’-3’) | Reverse (5’-3’) |
| --- | --- | --- |
| *G6pc1* | CCTCCTCAGCCTATGTCTGC | AACATCGGAGTGACCTTTGG |
| *Pck1* | GGGAACTCACTACTCGGGAA | GCCAGGTATTTCTTCTTGCC |
| *Pgc1α* | CCTACCGTTACACCTGTGAC | TACCTGCGCAAGCTTCTCTG |
| *Gcgr* | ATTGGCGATGACCTCAGTGTGA | GCAATAGTTGGCTATGATGCCG |
| *Pde1a* | ATGCAGCTGACGTCACTCAA | AGGGCCATGGTCCATCTGTA |
| *Pde2a* | TGGCGTTGTGGACGATGAG | CGCGATAGAAAAGCGGATGG |
| *Pde3a* | TCCCAGTCAGGAACCAGCAT | CAAGTTGCTTACGGCCCTC |
| *Pde3b* | AAAGCGCAGCCGGTTACTAT | CACCACTGCTTCAAGTCCCAG |
| *Pde4a* | TTTCCAACACGTTCCTAGACAAG | CCGGTGTGTACCAGCTTTTTC |
| *Pde4b* | GACTACATTGTCCATCCACTGTG | CTTGAGCATCCGGTTGAACCA |
| *Pde4c* | TGCTCAACCGTGAGTTGTCTT | GAAAGTCTGCGAGATGTACTCC |
| *Pde4d* | GGACAGTGGAAGTCAGGTGGAG | CGTATCAGGACAGCAGTCATCTGG |
| *Pde5a* | CGGCCTACCTGGCATTCTG | GCAAGGTCAAGTAACACCTGATT |
| *Pde6d* | CCCGTGTGCCCAAGAAAATC | CCACTCTTCTAGGCATTGTCCTT |
| *Pde7a* | AGTGGATCACCTCTAAGAGACG | CGGACATCTCCTAGCATACGAA T |
| *Pde7b* | TGCTAGGAGATGTACGACTAAG G | GGGCCTGCGGTATAATCCC |
| *Pde8a* | CCGAGCATCCACACTTCCG | TCAGCTACTGATACCTTCGAGG |
| *Pde9a* | CCACCATCTCCCTTTTAACCAC | CAGCACGCCCTGGATAAGT |
| *Pde11a* | AACAGGACCTACGATGAACAGG | TGAGGCAGATTCACCCTCGAT |
| *Cd36* | CCAAGCTATTGCGACATGATT | CCGAACACAGCGTAGATAGACC |
| *Ldlr* | TCAGTCCCAGGCAGCGTATC | CTTGATCTTGGCGGGTGTTC |
|  |  |  |
| *36b4* | CCAGCGAGGCCACACTGCTG | ACACTGGCCACGTTGCGGAC |
| *ß-actin* | TTCTTTGCAGCTCCTTCGTT | ATGGAGGGGAATACAGCCC |
| *Tbp* | ACCGTGAATCTTGGCTGTAAAC | GCAGCAAATCGCTTGGGATTA |
| *Hprt* | CCTAAGATGAGCGCAAGTTGAA | CCACAGGACTAGAACACCTGCTAA |

**Supplementary Table 2** Human oligonucleotide sequences used in this study.

| Gene | Forward (5’-3’) | Reverse (5’-3’) |
| --- | --- | --- |
| *ATP8B1* | TGGTGGATAGGACTGATGGTC | CGTTTACCAGGGCACCTTC |
| *PDE4D* | TGTGGCCTATCACAACAATATCC | CACAGCCAAATGATGGTTCTCTA |
| *CD36* | GAGAACTGTTATGGGGCTAT | TTCAACTGGAGAGGCAAAGG |
| *LDLR* | GTGTCACAGCGGCGAATG | CGCACTCTTTGATGGGTTCA |
|  |  |  |
| *36B4* | TCATCAACGGGTACAAACGA | GCCTTGACCTTTTCAGCAAG |
| *ß-ACTIN* | AGAGCTACGAGCTGCCTGAC | AGCACTGTGTTGGCGTACAG |
| *HPRT* | CCTGGCGTCGTGATTAGTGAT | AGACGTTCAGTCCTGTCCATAA |

**Supplementary Table 3** Antibodies used in this study.

| **Antibody** | **Manufacturer** | **Cat no.** |
| --- | --- | --- |
| Rabbit anti-ATP8B1 | Kindly provided by Dr. L.W. Klomp ^27^ |  |
| Rabbit anti-PDE4D | ProteinTech | 12918-1-AP |
| Rabbit anti-Akt | Cell Signaling | #4691 |
| Rabbit anti-p-Akt (Ser473) | Cell Signaling | #9271 |
| Rabbit anti-p-CREB (Ser133) | Cell Signaling | #9198 |
| Mouse-anti-Actin | ProteinTech | 66009-1-Ig |
| Rabbit-anti-GCGR | Abcam | ab75240 |
| Rabbit-anti-ATP1A1 | Kindly provided by Dr. J.B. Koenderink ^26^ |  |
| Rabbit-anti-GAPDH | Cell Signaling | #2118 |
