# Supplementary material for: ATP8B1 Deficiency Causes Phosphodiesterase 4‐Mediated Glucagon Resistance and Impaired Gluconeogenesis in Mouse and Human Liver
Source: Liver Int. 2025 Aug 25;45(9):e70306. doi: 10.1111/liv.70306 (PMC12375944; doi:10.1111/liv.70306)
Supplement: Supplementary file 2 — Figures S1–S4: liv70306‐sup‐0002‐FigureS1‐S4.docx. [file LIV-45-0-s002.docx]

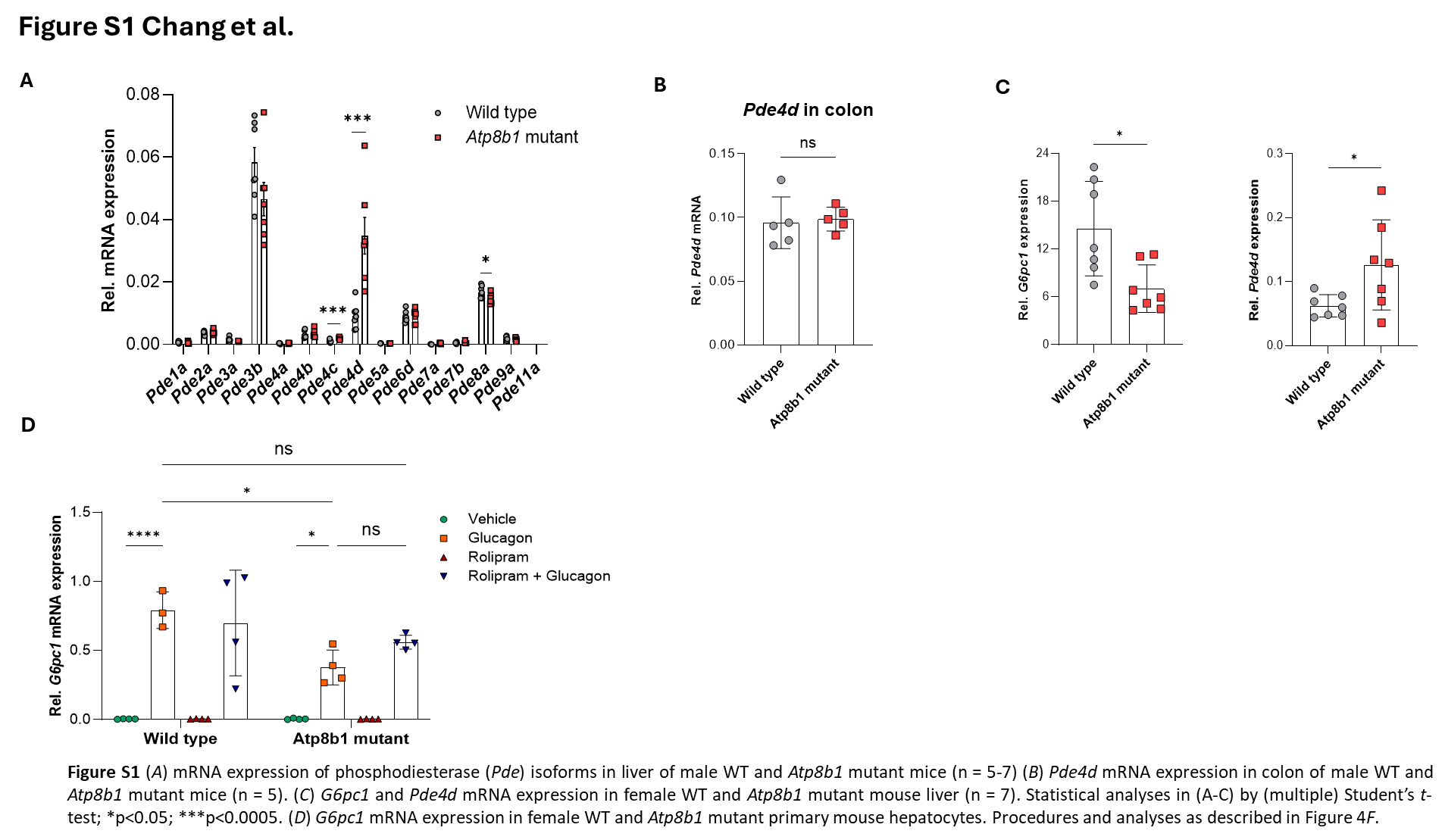


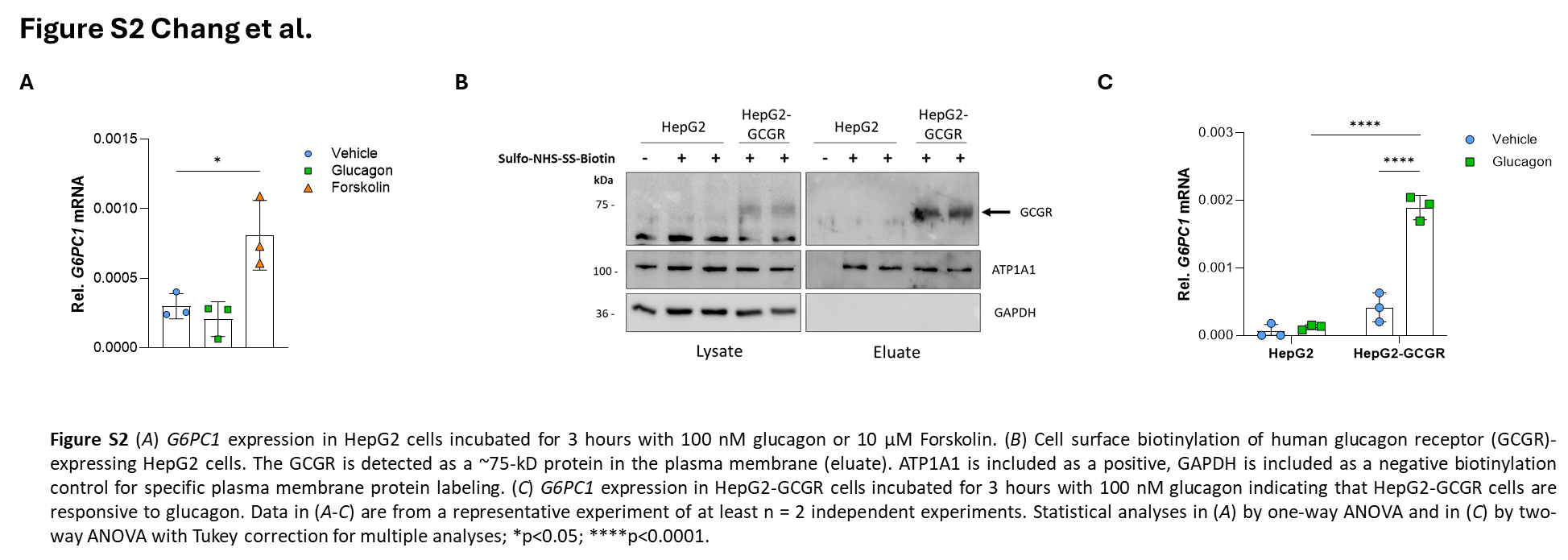


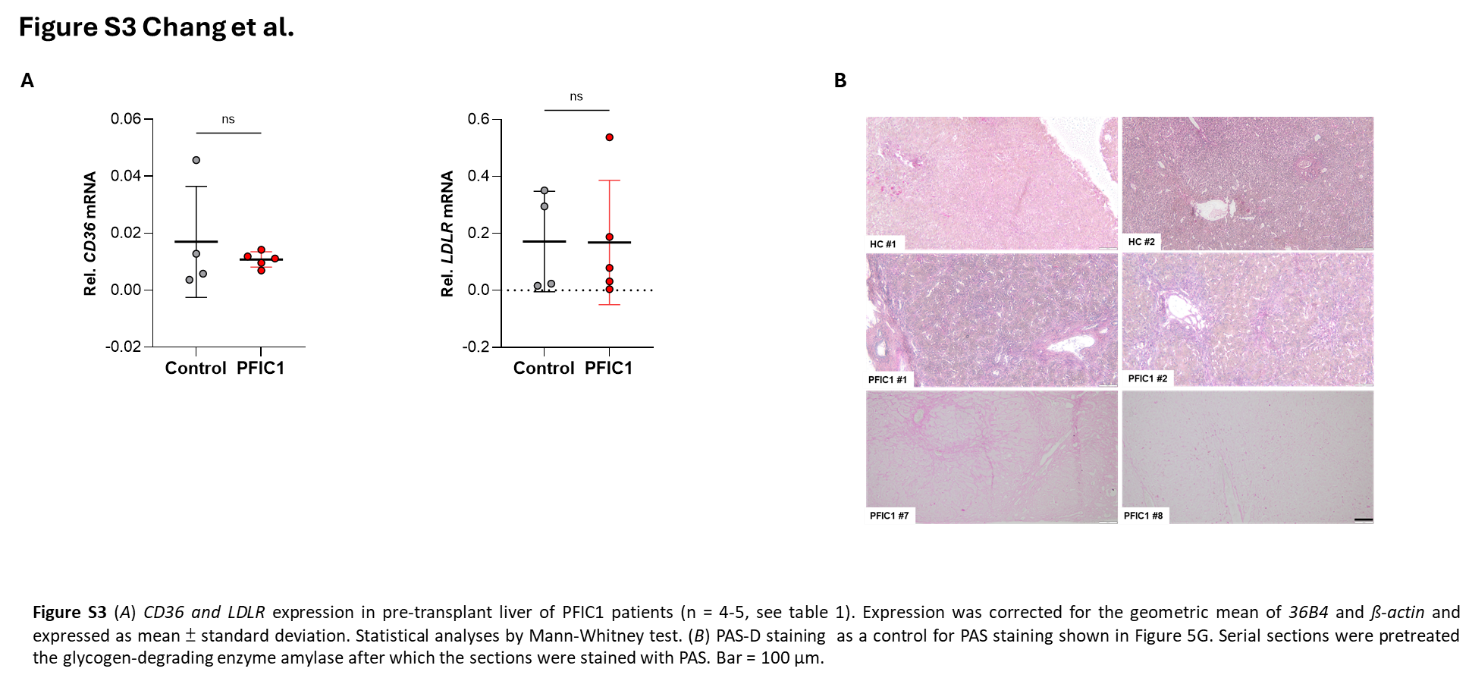


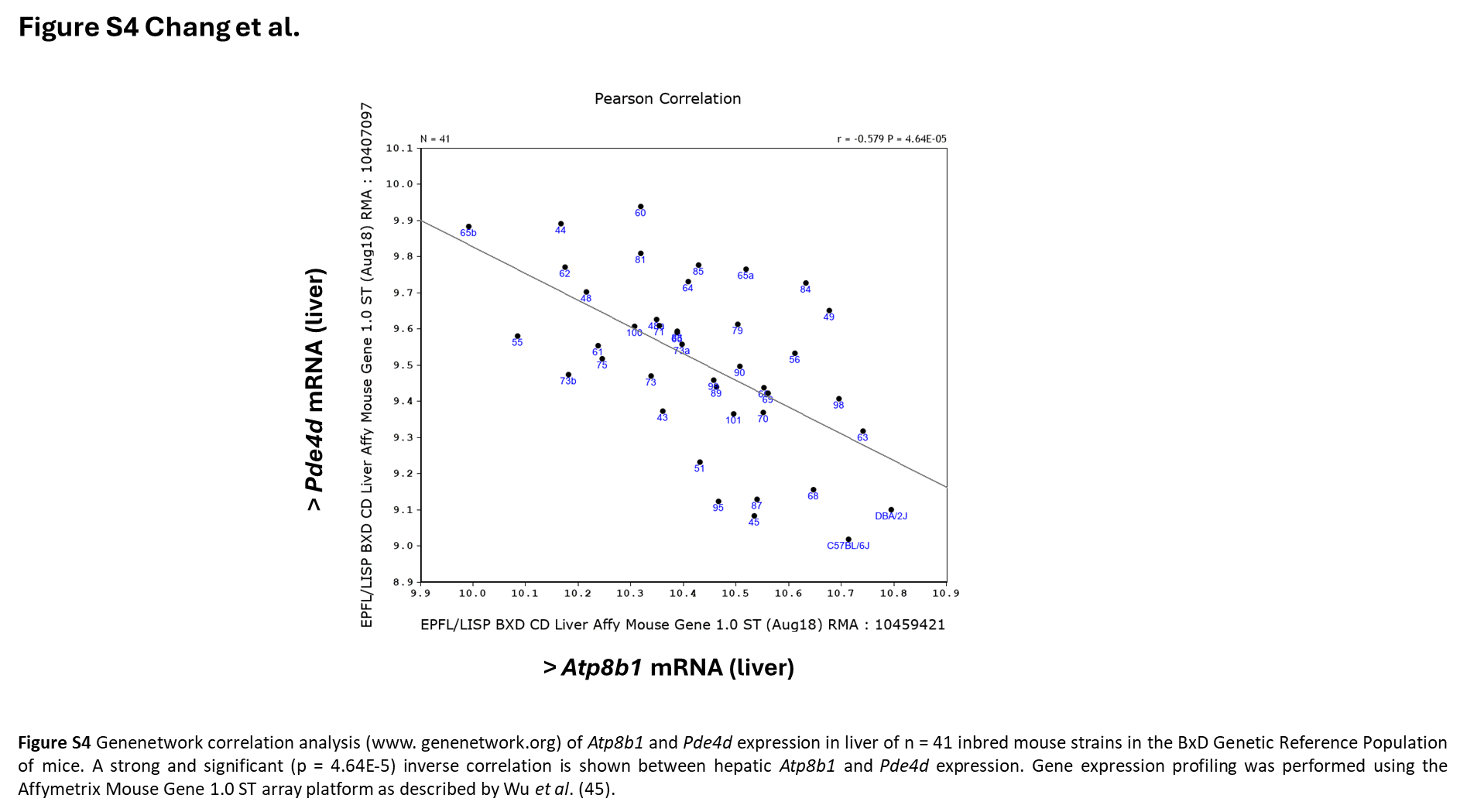


**Supplementary Table 1** Mouse oligonucleotide sequences used in this study.
**Supplementary Table 2** Human oligonucleotide sequences used in this study.
**Supplementary Table 3** Antibodies used in this study.
